# Association of CCL4 rs10491121 and rs1634507 gene polymorphisms with cancer susceptibility: trial sequential analysis and meta-analysis

**DOI:** 10.3389/fonc.2023.1133055

**Published:** 2023-08-01

**Authors:** Changsen Yang, Tiangang Song, Yajie Mo, Peixuan Wu, Haokun Tian, Lequan Wen, Yun Gao

**Affiliations:** ^1^ Joint Program of Nanchang University and Queen Mary University of London, Nanchang University, Nanchang, China; ^2^ The Second Affiliated Hospital of Nanchang University, Nanchang University, Nanchang, China; ^3^ Department of Physiology, Basic Medical College, Nanchang University, Nanchang, China

**Keywords:** CC cytokine ligand-4, rs10491121, rs1634507, single nucleotide polymorphisms, cancer

## Abstract

**Background:**

Although numerous case-control studies have explored the association between CC cytokine ligand-4 (CCL4) expression and cancer susceptibility, their results have been conflicting. This study aimed to determine the still-unknown connection of CCL4 rs10491121 and rs163450 polymorphisms with cancer susceptibility.

**Methods:**

Several databases, such as Web of Science, PubMed, and EMBASE, were searched for papers published since the creation of the database until November 2, 2022. Using RevMan 5.4 and StataMP 17 softwares, meta-analysis and subgroup analysis were performed after article screening and data extraction. For sensitivity analyses, one-by-one exclusion method was used, and then, the comprehensive effect was estimated and compared with that before exclusion. Trial sequential analysis (TSA)was performed using TSA 0.9.5.10 beta software.

**Results:**

Seven case-control studies encompassing 3559 cases and 4231 controls were included. The *P* value was greater than 0.05 for all models, indicating the absence of an evident relationship of CCL4 gene rs10491121 and rs1634507 polymorphisms with cancer susceptibility. However, in the subgroup analysis of rs10491121, the *P* values in all models studied by us except GA *vs.* AA were <0.05 considering the Chinese subgroup, suggesting that the G allele is a risk factor for cancer in the Chinese population. Besides, in the subgroup analysis of rs1634507 considering oral cancer, the co-dominant model GG *vs.* TT, dominant model GG + GT *vs.* TT, and allele model G *vs.* T groups showed OR < 1 and *P* < 0.05, indicating that the G allele was a protective factor of oral cancer. However, for other cancer types, all the models studied by us except GG *vs.* GT showed OR > 1 and *P* < 0.05, indicating that the G allele was a risk factor for these other cancers. Despite the statistically significant results, sensitivity analysis had some stability limitations, and TSA results suggested the possibility of false positives.

**Conclusion:**

For rs10491121, we identified an association between the G allele and increased cancer risk in the Chinese population. For rs1634507, the G allele was not found to be associated with reduced risk of oral cancer and increased risk of other cancers studied by us.

## 1 Introduction

C-C chemokine ligand (CCL) 4, also called macrophage inflammatory protein 1, belongs to the subfamily of CC chemokines and binds with various CC chemokine receptors (CCR) (1). The CC cytokine ligand-4 (CCL4) gene is located on chromosome 17, particularly q11-q21. Generally, CCL4 secretion is due to antigen stimulation or mitotic signals (2, 3). CCL4 can bind to CC chemokine receptor 5 (CCR5) and participate in various important processes. For example, CCL4 and CCR5 are crucial in atherosclerosis development (4) and in cancer development and immune system execution (5).

Among the many human gene mutation types, the most predominant are single nucleotide polymorphisms (SNPs), accounting for 90% of the gene mutations (6). SNPs introduce a difference of a single base in the DNA sequence (7). Thus far, many studies have explored the relationship of SNPs with cancer. For example, the SNP of rs763110 in the gene that encodes FasL will lead to changes in FasL expression such that increased FasL expression and decreased Fas expression will help cancer cells escape tumor immune response, leading to gynecological and other types of cancers (8). Besides, the SNP of the TLR3 gene at rs5743305 are suggested to be linked to increased breast cancer risk, whereas the SNP of the TLR3 gene at rs3775291 are reportedly linked to breast cancer recurrence (9). Furthermore, the SNP of the cadherin gene at rs9929218 can cause colorectal cancer (10). Therefore, SNPs can often contribute to the occurrence and development of cancer.

Notably, mutations at the two classical mutation sites of the CCL4 gene, namely rs1634507 and rs10491121, are reportedly closely related to many cancers. For example, rs1634507 has a certain role in predicting oral cavity carcinoma, and rs10491121 is closely associated with the tumor size of oral invasive squamous cell carcinoma (11). In addition, the SNP of rs10491121 is reportedly closely correlated with reduced hepatocellular carcinoma risk (12). In breast cancer patients, the probability of cancer metastasis to lymph nodes is lower in patients with the genotype AG or GG of rs10491121 than in those with genotype AA of rs10491121 (13). The risk of developing lung cancer is higher in individuals with genotype GT or TT of rs1634507 than in those with genotype AA of rs1634507 (14).

Evidently, there are many SNPs of rs1634507 and rs10491121, and different SNP combinations will lead to different results, particularly with respect to changes in cancer susceptibility. SNPs may aggravate or slow down the development and metastasis of cancer cells or may even not have any effect on cells of some cancer types. Recently, several case-control studies have been conducted on rs1634507 or rs10491121 polymorphisms and cancer risks. However, due to interference factors, the results of the abovementioned original studies have been conflicting, with some failing to show statistical significance. Therefore, the relationship of the SNPs of rs1634507 and rs10491121 with cancer is worth exploring using a meta-analysis.

## 2 Method

### 2.1 Study inclusion criteria

(1) Study content: to evaluate the correlation of cancer susceptibility with CCL4 SNPs at rs10491121 and rs1634507. (2) Study design: published case-control studies. (3) Participants: case group with pathologically and clinically diagnosed cancer patients; control group with general healthy population. Race, sex, age, and medical history were excluded from the analyses. (4) Original researches with reliable data quality, accurate application of statistical methods, and clear expression of results with available data on the number of people of each genotype in case and control groups.

### 2.2 Exclusion criteria

(1) Studies with incomplete data analysis or missing data wherein the author was unreachable to obtain the required information. (2) Non-human studies. (3) If the literature is repeatedly published by the same author, we selected the literature with the largest sample size.

### 2.3 Retrieval strategy

Web of Science, EMBASE, PubMed, CNKI, CBM, Wanfang, and VIP databases were searched for papers published since the creation of the database until November 2, 2022. A combination of subject words and free words was used in the retrieval process. The following were the terms searched: *CC cytokine ligand-4*, *cancer*, *CCL4*, *malignant tumor*, *carcinoma*, *lymphoma*, *neoplasm*, *variants*, and *polymorphism*. **Table 1** lists specific search strategies using PubMed as an example.

**Table 1:** The retrieval strategy using the PubMed database.

#1	CCL4
#2	CC cytokine ligand-4
#3	#1 OR #2
#4	cancer
#5	“carcinoma” [MeSH]
#6	malignant tumor
#7	neoplasm
#8	lymphoma
#9	#4 OR #5 OR #6 OR #7 OR #8
#10	polymorphism
#11	variants
#12	#10 OR #11
#13	#3 AND #9 AND #12

### 2.4 Article screening and data extraction

Two investigators independently extracted, screened, and cross-checked the literature. Problems encountered during article screening and data extraction were handled by consulting the third author. The existing literature was first screened by title. After excluding evidently irrelevant literature based on the title, the remaining literature was checked for validity by reading the Abstract and full text. If necessary, the original study author was contacted by phone or email to procure important information that was not provided in the publication. The data extraction process comprises several parts, including the basic information contained in the study, the year of publication, the first author of the study, study location, and the cancer type studied. Furthermore, it includes the number of pathologies contained in the case and control groups and the number of pathologies corresponding to each genotype.

### 2.5 Statistical methods

For meta-analysis, we used StataMP 17 and RevMan 5.4 were used in this study, and for trial sequential analysis (TSA), we used the TSA 0.9.5.10 beta software. The chi-square test was used for heterogeneity analyses (test level: α = 0.05). If no heterogeneity was identified in the results, the fixed effects model was used for meta-analysis. Conversely, if heterogeneity was identified in the results, a random effects model was adopted. The odds ratio (OR) value and 95% confidence interval (95% CI) of the allele as well as genotype frequency in each study were calculated. For subgroup analysis, the fixed effects model was used (15). A *P* value of <0.05 reflects statistical significance. In addition, subgroup analyses were performed by population and cancer type. We used the one-by-one exclusion method for sensitivity analyses, and the combined effects were estimated and then compared those before exclusion. We used the Begg’s and Egger’s tests to evaluate the publication bias of the included studies.

## 3 Result

### 3.1 Literature search and study characteristics

**Figure 1:**
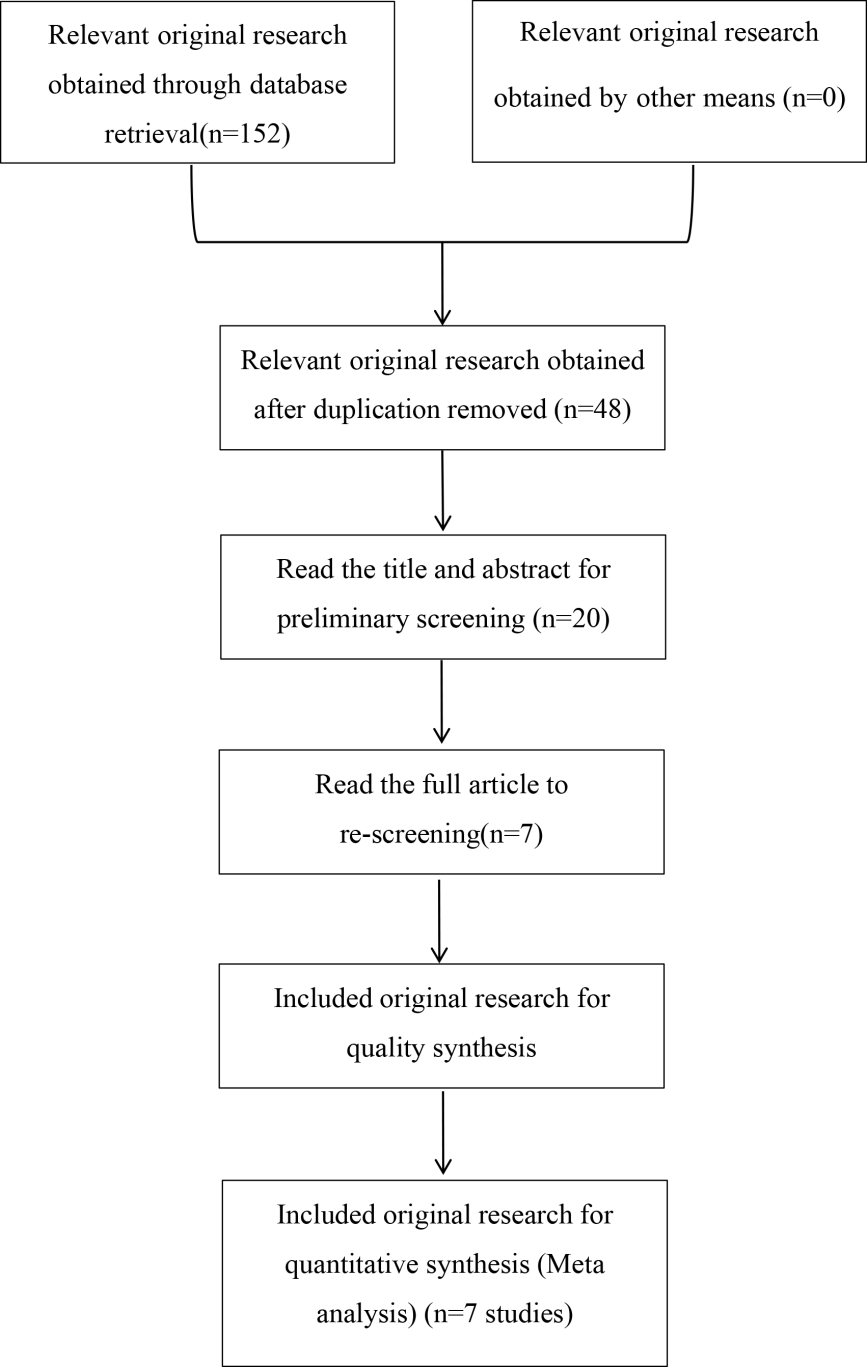
Study selection flow chart.

We retrieved a total of 152 relevant papers and finally included seven case-control studies after a stratified screening process. These seven studies encompassed 3559 cases and 4231 controls (11–14, 16–18). Among them, CCL4 rs10491121 polymorphisms were discussed in all seven studies and CCL4 rs1634507 polymorphisms were discussed in five case-control studies, encompassing 2109 cases and 3021 controls. Four studies were conducted on Chinese populations, and the other three studies on American, Iranian, and Swedish populations. **Figure 1** shows the literature selection process using PubMed as an example. **Tables 2** and **3** shows the basic characteristics of each included literature.

**Table 2:** Basic characteristics of included studies on the relationship between CCL4 rs10491121 polymorphism and cancer susceptibility.

Included study	Country	Number	Case groups			Control groups			Type of cancer
			GG	GA	AA	GG	GA	AA	
Bodelon C, 2013	USA	1641	298	406	136	288	368	145	Breast cancer
Lien M, 2017	China	2053	219	428	214	293	592	307	Oral cancer
Wang B, 2017	China	1546	91	152	103	296	609	295	Hepatocellular carcinoma
Hu G, 2018	China	523	83	152	79	53	92	64	Breast cancer
Hu W, 2020	China	908	133	272	133	45	165	160	Lung cancer
Shamoun L, 2021	Sweden	1019	250	281	79	176	181	52	Colorectal cancer
Kadeh H, 2022	Iran	100	25	18	7	26	20	4	Oral cancer

**Table 3:** Basic characteristics of included studies on the relationship between CCL4 rs1634507 polymorphism and cancer susceptibility.

Included study	Country	Number	Case groups			Control groups			Type of cancer
			GG	GA	AA	GG	GA	AA	
Lien M, 2017	China	2053	391	382	88	585	518	89	Oral cancer
Wang B, 2017	China	1546	167	148	31	575	517	108	Hepatocellular carcinoma
Hu G, 2018	China	523	135	138	41	101	83	25	Breast cancer
Hu W, 2020	China	908	213	242	83	94	175	101	Lung cancer
Kadeh H, 2022	Iran	100	23	24	3	30	19	1	Oral cancer

### 3.2 Meta-analysis results

#### 3.2.1 Relationship between rs10491121 polymorphism of the CCL4 gene and cancer susceptibility

**Figure 2:**
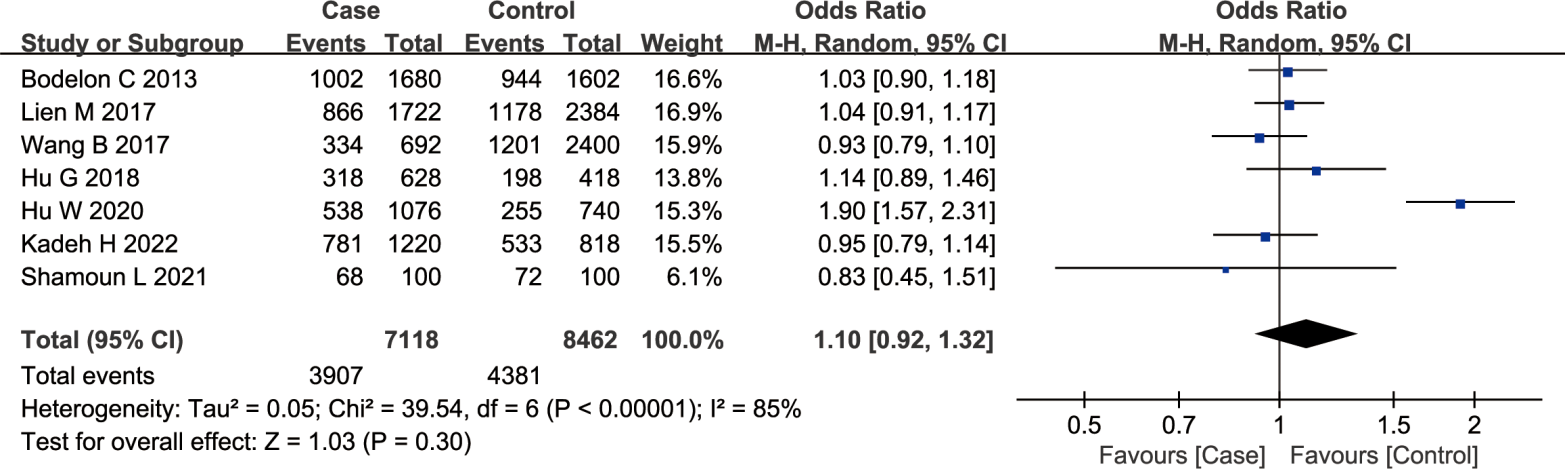
Meta-analysis of the correlation between rs10491121 polymorphism of the CCL4 gene and cancer susceptibility (G vs. A).

According to the findings of our meta-analysis, there were no significant differences in cancer susceptibility among the recessive model GG *vs.* GA + AA group [OR = 1.13, 95% CI (0.92, 1.39), P = 0.26, I^2^ = 70%], the dominant model GG + GA *vs.* AA group [OR = 1.14, 95% CI (0.85, 1.54), P = 0.37, I^2^ = 83%], and the co-dominant model GG *vs*. AA group [OR = 1.21, 95% CI (0.86, 1.72), P = 0.28, I^2^= 83%]. Furthermore, the OR values were as follows — GA *vs.* AA group: 1.12 [95% CI (0.85, 1.47), P = 0.41, I^2^= 77%], GG *vs.* GA group: 1.08 [95% CI (0.92, 1.26), P = 0.36, I2 = 44%], and G *vs.* A group: 1.10 [95% CI (0.92, 1.32), P = 0.30, I2 = 85%] (**Figure 2**). The *P* values in all of the above models were > 0.05, indicating no significant association of the rs10491121 G/A polymorphism of the CCL4 gene with cancer susceptibility.

#### 3.2.2 Relationship between rs1634507 polymorphism of the CCL4 gene and cancer susceptibility

**Figure 3:**
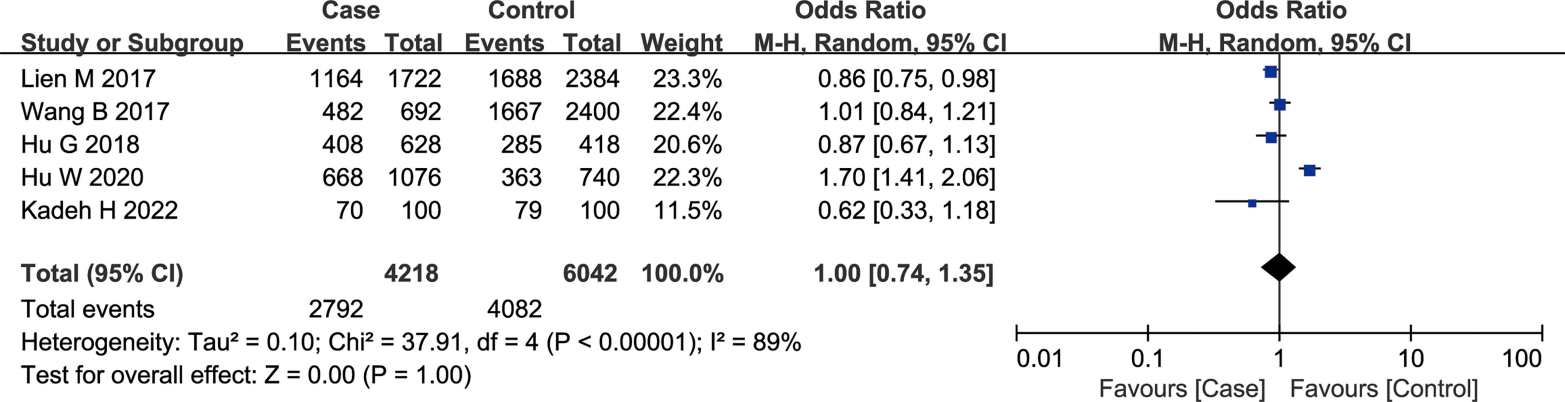
Meta-analysis of the correlation between rs1634507 polymorphism of the CCL4 gene and cancer susceptibility (G vs. T).

According to the findings of our meta-analysis, there were no significant differences in cancer susceptibility among the recessive model GG *vs.* GT + TT group [OR = 1.01, 95% CI (0.72, 1.41), P = 0.97, I^2^ = 84%], the dominant model GG + GT *vs.* TT group [OR = 1.03, 95% CI (0.61, 1.71), P = 0.92, I^2^ = 83%], and the co-dominant model GG *vs.* TT group [OR = 1.02, 95% CI (0.53, 1.97), P = 0.95, I^2^ = 88%]. Furthermore, the OR values were as follows — GT *vs.* TT group 1.04 [95% CI (0.71, 1.52), P = 0.84, I^2^ = 67%], GG *vs.* GT group: 1.00 [95% CI (0.77, 1.30), P = 0.98, I^2^ = 70%], and G *vs.* T group: 1.00 [95% CI (0.74, 1.35), P = 1.00, I^2^ = 89%] (**Figure 3**). The *P* values in all of the above models were > 0.05, indicating no significant association of the rs1634507 G/A polymorphism of the CCL4 gene with cancer susceptibility.

#### 3.2.3 Subgroup analysis

##### 3.2.3.1 Relationship between rs10491121 polymorphism of the CCL4 gene and cancer susceptibility

**Table 4** shows our findings of subgroup analyses of rs10491121 classified by country. The co-dominant model GG *vs.* AA [OR = 1.28, 95% CI (1.09, 1.51), P = 0.003]and GG *vs.* GA group [OR = 1.16, 95% CI (1.00, 1.35), P = 0.04], dominant model GG + GA *vs.* AA group [OR = 1.19, 95% CI (1.04, 1.35), P = 0.01], recessive model GG *vs.* GA+AA group [OR = 1.20, 95% CI (1.05, 1.38), P = 0.008], and allele model G *vs.* A group [OR = 1.15, 95% CI (1.05, 1.25), P = 0.001] suggested that the G allele was a risk factor for cancer in the Chinese population. However, this association was not significant in any genetic model for non-Chinese populations.

**Table 4:** Subgroup analysis of CCL4 rs10491121 polymorphism and cancer susceptibility.

Genetic contrasts	Group and subgroups	Studies (n)	Q test *P* value	I^2^	Model Selected	OR (95% CI)	*P*
GG *vs.* AA	Overall	7	*P* < 0.00001	83%	Random	1.21 (0.86, 1.72)	*P* = 0.28
	China	4	*P* < 0.00001	90%	Fixed	1.28 (1.09, 1.51)	*P* = 0.003
	Non-China	3	*P* = 0.53	0%	Fixed	1.02 (1.04, 1.36)	*P* = 0.84
GG + GA *vs*. AA	Overall	7	*P* < 0.00001	83%	Random	1.14 (0.85, 1.54)	*P* = 0.37
	China	4	*P* < 0.00001	91%	Fixed	1.19 (1.04, 1.35)	*P* = 0.01
	Non-China	3	*P* = 0.45	0%	Fixed	1.07 (0.87, 1.32)	*P* = 0.54
GG *vs*. GA + AA	Overall	7	*P* = 0.002	70%	Random	1.13 (0.92, 1.39)	*P* = 0.26
	China	4	*P* = 0.001	81%	Fixed	1.20 (1.05, 1.38)	*P* = 0.008
	Non-China	3	*P* = 0.93	0%	Fixed	0.95 (0.82, 1.11)	*P* = 0.55
GG *vs*. GA	Overall	7	*P* = 0.10	44%	Fixed	1.08 (0.92, 1.26)	*P* = 0.36
	China	4	*P* = 0.07	57%	Fixed	1.16 (1.00, 1.35)	*P* = 0.04
	Non-China	3	*P* = 0.94	0%	Fixed	0.93 (0.79, 1.10)	*P* = 0.41
GA *vs*. AA	Overall	7	*P* = 0.002	77%	Random	1.12 (0.85, 1.47)	*P* = 0.41
	China	4	*P* < 0.0001	88%	Fixed	1.13 (0.98, 1.30)	*P* = 0.09
	Non-China	3	*P* = 0.47	0%	Fixed	1.10 (0.88, 1.37)	*P* = 0.40
G *vs*. A	Overall	7	*P* < 0.00001	85%	Random	1.10 (0.92, 1.32)	*P* = 0.30
	China	4	*P* < 0.00001	91%	Fixed	1.15 (1.05, 1.25)	*P* = 0.001
	Non-China	3	*P* = 0.66	0%	Fixed	0.99 (0.89, 1.11)	*P* = 0.92

##### 3.2.3.2 Relationship between rs1634507 polymorphism of the CCL4 gene and cancer susceptibility

Subgroup analysis of rs1634507 classified by cancer type was performed (**Table 5**). The allele model G *vs.* T group [OR = 0.85, 95% CI (0.74, 0.97), P = 0.01], dominant model GG + GT *vs.* TT group [OR = 0.70, 95% CI (0.51, 0.95), P = 0.02], and co-dominant model GG *vs.* TT group [OR = 0.66, 95% CI (0.48, 0.91), P = 0.01] revealed the G gene as a protective factor of oral cancer. However, for other cancer types studied by us, the dominant model GG + GT *vs.* TT group [OR = 1.41, 95% CI (1.11, 1.77), P = 0.004], co-dominant model GG *vs.* TT group [OR = 1.51, 95% CI (1.18, 1.95), P = 0.001], recessive model GG *vs.* GT + TT group [OR = 1.19, 95% CI (1.01, 1.40), P = 0.04], and GT *vs.* TT group [OR = 1.30, 95% CI (1.01, 1.66), P = 0.04] and allele model G *vs.* A group [OR = 1.20, 95% CI (1.06, 1.35), P = 0.003] suggested that presence of the G allele as a risk factor for cancer.

**Table 5:** Subgroup analysis of CCL4 rs1634507 polymorphism and cancer susceptibility.

Genetic contrasts	Group and subgroups	Studies (n)	Q test *P* value	I^2^	Model Selected	OR (95% CI)	*P*
GG *vs*. TT	Overall	5	*P <* 0.00001	88%	Random	1.02 (0.53, 1.97)	*P =* 0.95
	Oral cancer	2	*P =* 0.42	0%	Fixed	0.66 (0.48, 0.91)	*P =* 0.01
	Non-oral cancer	3	*P =* 0.0001	89%	Fixed	1.51 (1.18, 1.95)	*P =* 0.001
GG + GT *vs*. TT	Overall	5	*P <* 0.0001	83%	Random	1.03 (0.61, 1.71)	*P =* 0.92
	Oral cancer	2	*P =* 0.50	0%	Fixed	0.70 (0.51, 0.95)	*P =* 0.02
	Non-oral cancer	3	*P =* 0.006	81%	Fixed	1.41 (1.11, 1.77)	*P =* 0.004
GG *vs*. GT + TT	Overall	5	*P <* 0.0001	84%	Random	1.01 (0.72, 1.41)	*P =* 0.97
	Oral cancer	2	*P =* 0.31	2%	Fixed	0.85 (0.71, 1.00)	*P =* 0.06
	Non-oral cancer	3	*P =* 0.0002	88%	Fixed	1.19 (1.01, 1.40)	*P =* 0.04
GG *vs*. GT	Overall	5	*P =* 0.009	70%	Random	1.00 (0.77, 1.30)	*P =* 0.98
	Oral cancer	2	*P =* 0.34	0%	Fixed	0.89 (0.74, 1.06)	*P =* 0.20
	Non-oral cancer	3	*P =* 0.009	79%	Fixed	1.12 (0.94, 1.33)	*P =* 0.19
GT *vs*. TT	Overall	5	*P =* 0.02	67%	Random	1.04 (0.71, 1.52)	*P =* 0.84
	Oral cancer	2	*P =* 0.64	0%	Fixed	0.74 (0.54, 1.01)	*P =* 0.06
	Non-oral cancer	3	*P =* 0.12	53%	Fixed	1.30 (1.01, 1.66)	*P =* 0.04
G *vs*. T	Overall	5	*P <* 0.00001	89%	Random	1.00 (0.74, 1.35)	*P =* 1.00
	Oral cancer	2	*P =* 0.33	0%	Fixed	0.85 (0.74, 0.97)	*P =* 0.01
	Non-oral cancer	3	*P <* 0.0001	91%	Fixed	1.20 (1.06, 1.35)	*P =* 0.003

#### 3.2.4 Sensitivity analysis

Taking the rs10491121 group GG + GA over AA as an example, the minimum and maximum combined ORs were 1.01 [95% CI (0.86, 1.19)] and 1.24 [95% CI (0.91, 1.69)], respectively, after excluding one study. Sensitivity analyses indicated that meta-analysis results were vulnerable to changing significantly from the inclusion or exclusion of a single study.

#### 3.2.5 Publication bias analysis

**Figure 4:**
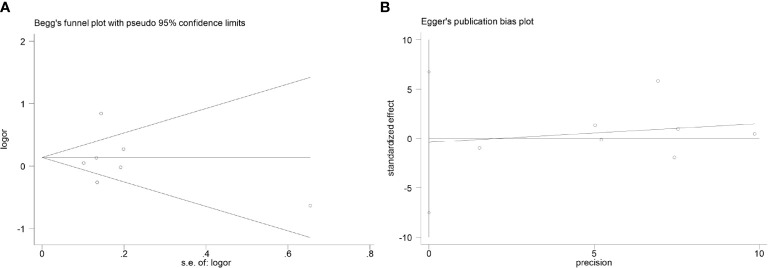
Publication bias analysis results (considering the GG + GA vs. AA genotype of rs10491121 and cancer susceptibility as an example). **(A)**Begg's test. **(B)** Egger's test.

**Tables 6** and **7** show the results of publication bias analyses of CCL4 rs10491121 and rs1634507 polymorphisms. After the Begg’s test, we drew a funnel plot of GG + GA *vs.* AA genotypes at the locus rs10491121. The seven studies included appeared completely on the chart, distributed around the combined OR value. The pattern showed a symmetrical trend and an inverted funnel shape, indicating the absence of publication bias. Because few studies with single SNP sites were included in the meta-analysis, quantitative findings of the Begg’s test were Z = 0.00 and *P* = 1.000 (>0.05). Findings of the Egger’s test were t = -0.14, P = 0.896 (>0.05), and 95% CI of -7.52 to 6.75, suggesting no publication bias (**Figure 4**).

**Table 6:** Publication bias analysis of the rs10491121 gene polymorphism of CCL4 and cancer susceptibility.

Genotype	Egger's test				Begg's Test	
	Std. Err.	t	*P*	95% CI	Z	*P*
GG *vs*. AA	2.80	0.19	0.854	(-6.66, 7.74)	0.30	0.764
(GG + GA) *vs*. AA	2.78	-0.14	0.896	(-7.52, 6.75)	0.00	1.000
GG *vs*. (GA + AA)	2.11	0.76	0.480	(-3.81, 7.02)	0.60	0.548
G *vs*. A	3.10	0.22	0.837	(-7.29, 8.64)	0.00	1.000

**Table 7:** Publication bias analysis of the rs1634507 gene polymorphism of CCL4 and cancer susceptibility.

Genotype	Egger's test				Begg's Test	
	Std. Err.	t	*P*	95% CI	Z	*P*
GG *vs*. TT	3.6	-0.27	0.804	(-12.43, 10.48)	0.24	0.806
(GG + GT) *vs*. TT	2.91	-0.44	0.687	(-10.56, 7.97)	0.24	0.806
GG *vs*. (GT + TT)	3.42	-0.01	0.99	(-10.94, 10.85)	-0.24	1.000
G *vs*. T	4.14	-0.11	0.918	(-13.64, 12.71)	-0.24	1.000

#### 3.2.6 Trial sequential analysis

**Figure 5:**
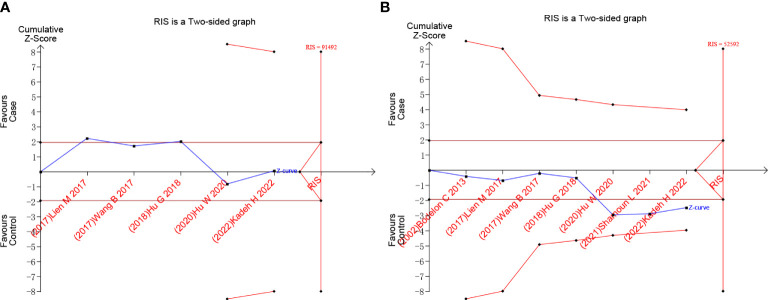
Trial sequential analysis results of G vs. A of the rs10491121 group and G vs. T of rs1634507 group. **(A)** G vs. A of rs10491121. **(B)** G vs. T of rs1634507.

We used TSA to calculate the sample size required to draw definitive conclusions and analyze random error problems in repeated updates of meta-analyses, such as false negatives and false positives. Taking the example of rs10491121 G *vs.* A, the accumulated information size is too small to reach the required information size, and the Z curve tends to almost intersect with the TSA boundary (**Figure 5A**). As shown in **Figure 5B**, the result of rs1634507 G *vs.* T group also had a similar tendency. The results revealed that the possibility of false positives still persisted (19). Therefore, to further verify this result, more follow-up case-control studies are warranted.

## 4 Discussion

Our results showed that cancer susceptibility was not significantly differently exacerbated by the polymorphisms of CCL4 gene at rs10491121 and rs1634507 at the macroscopic level. However, subgroup analysis revealed an association between the G allele and increased cancer risk for rs10491121 in the Chinese population. For rs1634507, there was an association between the G allele and reduced risk of oral cancer and increased risk of other cancers studied by us.

CCL4 is also known as macrophage inflammatory protein 1 beta (2). It can bind to CCR5, a seven-transmembrane G protein coupled receptor, on the cell surface (3). CCL4 is essential for inflammation, tumorigenesis, and other immune responses, particularly tumor growth, metastasis, angiogenesis, and invasion (20–23), thus making it closely related to the pathogenesis of various cancers (14). CCL-4 expression reportedly increases on the surface of prostate cancer cells, which causes changes in the integrin pathway and exacerbates the development and metastasis of prostate cancer. It can also accelerate prostate cancer development *via* signal translator and activator of transfer 3 (STAT3)-dependent signal transduction (24). Besides, tumor-infiltrating granulocytes and monocellular myeloid-derived suppressor cells can release CCL4 in surplus to act on CCR5 receptors on melanoma and lymphoma cancer cells, thus recruiting a large number of T regulatory cells and promoting the generation, growth, and metastasis of cancer (25). In addition, the CCL4–CCR5 interaction can regulate the interaction of fibroblasts with cancer cells in the bone cavity to promote bone metastasis of breast cancer (26). Serum CCL4 levels are reportedly significantly higher in patients with squamous cell carcinoma of the head and neck than in controls. The situation is observed in patients with hepatocellular carcinoma (HCC) (11). However, in patients with esophageal squamous cell carcinoma, the increased CCL4 expression may also improve the prognosis by altering the cancer microenvironment and recruiting CD8+ T cells to strengthen cancer immunity (27). In the case of inflammation, CCL4 secretion will be promoted by CD4+ cells forming a complex with dendritic cells and antigens. CCL4 release promotes the interaction between CD4+ cells and the CCR5 receptors present on CD8+ cells. This process is associated with increased cross initiation between lymphocytes; this can lead to CD8+ T lymphocytes flowing out of lymph nodes and reaching the cancer cells, providing a strong and long-term immune response (18, 28). In this way, increased CCL4 expression may lower the risk of cancer and provide a better prognosis. For example, for esophageal squamous cell carcinoma patients, a high CCL4 level indicates prolonged survival (27). However, there is a paucity of research on the relationship between CCL4 levels and cancer susceptibility, possibly because it is difficult to measure CCL4 levels in the “carcinogenic niche,” which comprises inflammatory cells and their released cytokines in the tumor microenvironment. Genetically determined differences in cytokine and chemokine gene transcript levels have been identified and demonstrated between tumor-susceptible and -resistant animal models (29). Rs1634507 is located in the promoter region of CCL4 (12), and its mutation markedly affects CCL4 expression. Therefore, it is reasonable to speculate that polymorphisms that cause changes in expression level and activity contribute of CCL4 to cancer susceptibility in patients. Additionally, CCL4 expression level affects different cancers differently. The differences in characteristics of this mutation in oral cancer and other cancers studies by us may be attributed to different gene expression levels affecting different cancers differently, and a better understanding in this regard warrants further exploration.

CCL4 interaction with CCR5 can enhance the anti-tumor immune effect by making γδT cells enter cancer tissues from peripheral blood (30). HCC-C2 is a type of hepatocellular carcinoma that particularly affects Asians and occurs in Chinese and Asian American individuals but not in Europeans. γδT cells are highly expressed in this type of liver cancer (31). Therefore, the mutation of rs10491121 possibly affects γδT cell recruitment by CCL4, thus causing a higher level of γδT cells in Chinese individuals with cancer and consequently reducing cancer susceptibility and improving tumor immunity of Chinese individuals. However, to gain a better understanding of the potential carcinogenic and anti-tumor mechanisms of rs10491121, more pertinent laboratory researches are warranted required. Furthermore, the discovery that capecitabine can reduce CTLA-4 expression in CRC cells suggests that traditional chemotherapy can influence the immune response (32), which may also apply to CCL4. Therefore, further research into the relationship between CCL4 polymorphism and the efficacy of standard anticancer therapy is warranted.

Begg’s and Egger’s test findings revealed no publication bias in this study, thus upholding the credibility of our results. Despite this, the study has some limitations. First, all studies involved herein were from published articles written in English or Chinese. Therefore, our results may be prone to a language bias. Second, given that rs10491121 and rs1634507 polymorphisms of the CCL4 gene and cancer susceptibility are not widely studied topics, few studies were available for reference, thus limiting the number of included cases and controls and the number of subgroup analysis studies and affecting the representativeness of the findings, as shown by the TSA results. Furthermore, the scope of the study was limited to published case-control studies, and studies with incomplete or missing data analysis were excluded. This selection criterion may have resulted in a smaller sample size, thus lowering the statistical power of the meta-analysis. Besides, confounding factors like race, gender, age, and medical history were not considered in the study. These variables may have influenced the relationship between cancer susceptibility and CCL4 SNPs. Consequently, although the study does offer significant insights into the association between CCL4 SNPs and cancer susceptibility, caution should be exercised when interpreting the findings. Further studies are required to validate and confirm these results.

Taken together, according to the above results, cancer susceptibility was not significantly differently exacerbated by the polymorphisms of CCL4 gene at rs10491121 and rs1634507 at the macroscopic level. However, subgroup analysis revealed that for rs10491121, an association of the G allele with increased cancer risk existed in the Chinese population. For rs1634507, the G allele was found to be associated with reduced risk of oral cancer and increased risk of other cancers studied by us. For a more informative meta-analysis, more multi-center case-control studies with large sample sizes are warranted in the future.

## Data availability statement

The raw data supporting the conclusions of this article will be made available by the authors, without undue reservation.

## Author contributions

YG was responsible for the entire project and revised the draft. CY, TS, YM, PW, HT, and LW performed the systematic review and drafted the first version of the manuscript. All authors participated in the interpretation of the results and prepared the final version of the manuscript.

## References

[1] Oliveira SH Lira S Martinez AC Wiekowski M Sullivan L Lukacs NW . Increased responsiveness of murine eosinophils to Mip-1beta (Ccl4) and Tca-3 (Ccl1) is mediated by their specific receptors, Ccr5 and Ccr8. J Leukoc Biol (2002) 71(6):1019–25. doi: 10.1189/jlb.71.6.1019 12050188

[2] Menten P Wuyts A Van Damme J . Macrophage inflammatory protein-1. Cytokine Growth Factor Rev (2002) 13(6):455–81. doi: 10.1016/s1359-6101(02)00045-x 12401480

[3] Maurer M von Stebut E . Macrophage inflammatory protein-1. Int J Biochem Cell Biol (2004) 36(10):1882–6. doi: 10.1016/j.biocel.2003.10.019 15203102

[4] Chang TT Yang HY Chen C Chen JW . Ccl4 inhibition in atherosclerosis: effects on plaque stability, endothelial cell adhesiveness, and macrophages activation. Int J Mol Sci (2020) 21(18):6567. doi: 10.3390/ijms21186567 32911750 PMC7555143

[5] Korbecki J Grochans S Gutowska I Barczak K Baranowska-Bosiacka I . Cc chemokines in a tumor: a review of pro-cancer and anti-cancer properties of receptors Ccr5, Ccr6, Ccr7, Ccr8, Ccr9, and Ccr10 ligands. Int J Mol Sci (2020) 21(20):7619. doi: 10.3390/ijms21207619 33076281 PMC7590012

[6] Bernig T Chanock SJ . Challenges of Snp genotyping and genetic variation: its future role in diagnosis and treatment of cancer. Expert Rev Mol Diagn (2006) 6(3):319–31. doi: 10.1586/14737159.6.3.319 16706736

[7] Lee JE . High-throughput genotyping. Forum Nutr (2007) 60:97–101. doi: 10.1159/000107078 17684405

[8] Zhou L Zhang G Zhou X Li J . The association between the Snp Rs763110 and the risk of gynecological cancer: a meta-analysis. BioMed Pharmacother (2015) 69:208–13. doi: 10.1016/j.biopha.2014.11.022 25661359

[9] Tian H Xu W Wen L Tang L Zhang X Song T . Association of Tlr3 gene 1377c/T (Rs3775290) and Tlr7 gene C/G (Rs3853839) polymorphism with hand, foot, and mouth disease caused by human Enterovirus 71 infection susceptibility and severity in the chinese han population: a meta-analysis of case-control studies. Med (Baltimore) (2022) 101(27):e29758. doi: 10.1097/md.0000000000029758 PMC9259132 35801751

[10] Wang H Gu D Yu M Hu Y Chen Z Huo X . Variation Rs9929218 and risk of the colorectal cancer and adenomas: a meta-analysis. BMC Cancer (2021) 21(1):190. doi: 10.1186/s12885-021-07871-z 33627078 PMC7903630

[11] Lien MY Lin CW Tsai HC Chen YT Tsai MH Hua CH . Impact of Ccl4 gene polymorphisms and environmental factors on oral cancer development and clinical characteristics. Oncotarget (2017) 8(19):31424–34. doi: 10.18632/oncotarget.15615 PMC5458219 28404909

[12] Wang B Chou YE Lien MY Su CM Yang SF Tang CH . Impacts of Ccl4 gene polymorphisms on hepatocellular carcinoma susceptibility and development. Int J Med Sci (2017) 14(9):880–4. doi: 10.7150/ijms.19620 PMC5562195 28824325

[13] Hu GN Tzeng HE Chen PC Wang CQ Zhao YM Wang Y . Correlation between Ccl4 gene polymorphisms and clinical aspects of breast cancer. Int J Med Sci (2018) 15(11):1179–86. doi: 10.7150/ijms.26771 PMC6097259 30123055

[14] Hu W Chien SY Ying P Liu PI Su CM Tang CH . Impact of Ccl4 gene polymorphisms upon the progression of lung cancer in a han chinese cohort. Med (Baltimore) (2020) 99(3):e18906. doi: 10.1097/md.0000000000018906 PMC7220213 32011520

[15] Tufanaru C Munn Z Stephenson M Aromataris E . Fixed or random effects meta-analysis? common methodological issues in systematic reviews of effectiveness. Int J Evidence-Based Healthc (2015) 13(3):196–207. doi: 10.1097/xeb.0000000000000065 26355603

[16] Bodelon C Malone KE Johnson LG Malkki M Petersdorf EW McKnight B . Common sequence variants in chemokine-related genes and risk of breast cancer in post-menopausal women. Int J Mol Epidemiol Genet (2013) 4(4):218–27. PMC3852641 24319537

[17] Shamoun L Landerholm K Balboa Ramilo A Andersson RE Dimberg J Wågsäter D . Association of gene and protein expression and genetic polymorphism of Cc chemokine ligand 4 in colorectal cancer. World J Gastroenterol (2021) 27(30):5076–87. doi: 10.3748/wjg.v27.i30.5076 PMC8384737 34497436

[18] Kadeh H Eyni M Parsasefat M Miri-Moghaddam E . The Association of Ccl4 Rs1634507 and Rs10491121 Polymorphisms with Susceptibility of Oral Squamous Cell Carcinoma in an Iranian population: a case-control study. Iranian J Pathol (2022) 17(2):210–6. doi: 10.30699/ijp.2022.538948.2725 PMC9013867 35463731

[19] Tian H Xu W Wen L Tang L Zhang X Song T . Association of Ptpn22 Snp1858 (Rs2476601) and Gene Snp1123 (Rs2488457) polymorphism with primary immune thrombocytopenia susceptibility: a meta-analysis of case-control studies and trial sequential analysis. Front Genet (2022) 13:893669. doi: 10.3389/fgene.2022.893669 35692826 PMC9174638

[20] Lien MY Tsai HC Chang AC Tsai MH Hua CH Wang SW . Chemokine Ccl4 induces vascular endothelial growth factor C expression and lymphangiogenesis by Mir-195-3p in oral squamous cell carcinoma. Front Immunol (2018) 9:412. doi: 10.3389/fimmu.2018.00412 29599774 PMC5863517

[21] Mollica Poeta V Massara M Capucetti A Bonecchi R . Chemokines and chemokine receptors: new targets for cancer immunotherapy. Front Immunol (2019) 10:379. doi: 10.3389/fimmu.2019.00379 30894861 PMC6414456

[22] Sivina M Werner L Rassenti L Ferrajoli A Wierda WG Keating MJ . Dynamic changes in Ccl3 and Ccl4 plasma concentrations in patients with chronic lymphocytic leukaemia managed with observation. Br J Haematol (2018) 180(4):597–600. doi: 10.1111/bjh.14398 27766619 PMC7021206

[23] Wang Y Liu T Yang N Xu S Li X Wang D . Hypoxia and macrophages promote glioblastoma invasion by the Ccl4-Ccr5 axis. Oncol Rep (2016) 36(6):3522–8. doi: 10.3892/or.2016.5171 27748906

[24] Fang LY Izumi K Lai KP Liang L Li L Miyamoto H . Infiltrating macrophages promote prostate tumorigenesis *via* modulating androgen receptor-mediated Ccl4-Stat3 signaling. Cancer Res (2013) 73(18):5633–46. doi: 10.1158/0008-5472.Can-12-3228 PMC3833080 23878190

[25] Schlecker E Stojanovic A Eisen C Quack C Falk CS Umansky V . Tumor-infiltrating monocytic myeloid-derived suppressor cells mediate Ccr5-dependent recruitment of regulatory T cells favoring tumor growth. J Immunol (2012) 189(12):5602–11. doi: 10.4049/jimmunol.1201018 23152559

[26] Sasaki S Baba T Nishimura T Hayakawa Y Hashimoto S Gotoh N . Essential roles of the interaction between cancer cell-derived chemokine, Ccl4, and intra-bone Ccr5-expressing fibroblasts in breast cancer bone metastasis. Cancer Lett (2016) 378(1):23–32. doi: 10.1016/j.canlet.2016.05.005 27177471

[27] Liu JY Li F Wang LP Chen XF Wang D Cao L . Ctl- Vs Treg lymphocyte-attracting chemokines, Ccl4 and Ccl20, are strong reciprocal predictive markers for survival of patients with oesophageal squamous cell carcinoma. Br J Cancer (2015) 113(5):747–55. doi: 10.1038/bjc.2015.290 PMC4559838 26284335

[28] González-Martín A Mira E Mañes S . Ccr5 in cancer immunotherapy: more than an “attractive” receptor for T cells. Oncoimmunology (2012) 1(1):106–8. doi: 10.4161/onci.1.1.17995 PMC3376953 22720226

[29] Okada F Izutsu R Goto K Osaki M . Inflammation-related carcinogenesis: lessons from animal models to clinical aspects. Cancers (Basel) (2021) 13(4):921. doi: 10.3390/cancers13040921 33671768 PMC7926701

[30] Zhao N Dang H Ma L Martin SP Forgues M Ylaya K . Intratumoral Γδ T-Cell Infiltrates, Chemokine (C-C Motif) ligand 4/Chemokine (C-C Motif) ligand 5 protein expression and survival in patients with hepatocellular carcinoma. Hepatology (2021) 73(3):1045–60. doi: 10.1002/hep.31412 PMC9175512 32502310

[31] Chaisaingmongkol J Budhu A Dang H Rabibhadana S Pupacdi B Kwon SM . Common molecular subtypes among Asian hepatocellular carcinoma and Cholangiocarcinoma. Cancer Cell (2017) 32(1):57–70.e3. doi: 10.1016/j.ccell.2017.05.009 28648284 PMC5524207

[32] Derakhshani A Hashemzadeh S Asadzadeh Z Shadbad MA Rasibonab F Safarpour H . Cytotoxic T-Lymphocyte Antigen-4 in colorectal cancer: another therapeutic side of Capecitabine. Cancers (Basel) (2021) 13(10):2414. doi: 10.3390/cancers13102414 34067631 PMC8155910

